# Routine liver ultrasound screening does not alter clinical management in a cohort study of multiple cutaneous infantile haemangioma

**DOI:** 10.1111/bjd.19472

**Published:** 2020-12-28

**Authors:** C. Mahon, K. McHugh, N. Alband, D. Rampling, N. Sebire, E. Williamson, M. Glover, V.A. Kinsler

**Affiliations:** ^1^ Paediatric Dermatology Great Ormond Street Hospital for Children London UK; ^2^ Paediatric Radiology Great Ormond Street Hospital for Children London UK; ^3^ Paediatric Pathology Great Ormond Street Hospital for Children London UK; ^4^ UCL Great Ormond Street Institute of Child Health London UK; ^5^ The Francis Crick Institute London UK

dear editor, Cutaneous infantile haemangioma (IH) occurs in 5–10% of neonates, with multiple IH (> 1) in 30% of those.^1^ Expert opinion currently recommends routine liver ultrasound (USS) for five or more (5+) IH,^2^, ^3^ and if hepatic haemangiomas (HH) are identified, to test thyroid function^3^ even if neonatal thyroid screening occurred. Supportive data from one cohort demonstrate associations between 5+ IH and HH,^1^ and between HH and adverse clinical outcomes (cardiac failure/hypothyroidism).^4^, ^5^, ^6^ However, there are no data relating number of cutaneous IH, USS screening or thyroid testing to frequency of adverse clinical outcomes, as per the principles of any screening programme.^7^ One study suggesting validation of screening USS in infants with multiple cutaneous IH, while important in describing a HH cohort, in fact compared clinical outcomes in infants presenting spontaneously (in whom we would expect more severe disease) with those diagnosed on screening.^4^ In addition, the study lacked control groups without USS and without HH.

Therefore, three fundamental questions remain: (i) is there is a statistically significant association between number of cutaneous IH and adverse clinical outcome; (ii) does screening USS alter rates of adverse clinical outcome; and (iii) does thyroid function retesting of those with HH alter clinical outcome. While this is a large study and every attempt has been made to collect and analyse data accurately, a prospective randomized control trial will be the only way to answer questions (ii) and (iii) definitively.

Two retrospective cohorts were identified from an electronic patient records search, and combined: infants with multiple IH (seen 1996–2016), and solitary IH (2015–2016), to assess question (i). As screening advice changed over 20 years, we were able to include infants with and without liver USS and repeat thyroid testing, to assess questions (ii) and (iii). Infants with PHACE syndrome were excluded, and no patient had multifocal lymphangiomatosis with thrombocytopenia.^8^ Median age at presentation to our department with IH was 0·50 years (mean 0·92 years; SEM 0·05), with 0·47 years for the subset with multiple IH (mean 0·65 years; SEM 0·03), and 0·21 years for those with cardiac failure (mean 0·33 years; SEM 0·08). Median age at first USS in our hospital was 0·39 years (mean 0·56 years; SEM 0·04).

Of 843 infants studied, 74% were female; 83% had multiple IH. There were no deaths. Of the 381 screened by USS, 71 (19%) had HH (10 single, 58 multiple, two diffuse and one multiple‐almost‐diffuse), of which more than a third (24 of 71) had < 5 IH and would have been missed by current screening recommendations. Despite the high rate of HH, < 1% (eight of 843) required treatment for cardiac failure and < 1% (six of 843) for hypothyroidism (one patient requiring treatment for both), in line with previous reports.^3^ Six of 13 requiring treatment had < 5 IH, and eight of 13 had HH.

Overall, on regression analysis with Bonferroni correction, the number of cutaneous IH was not significantly associated with treatment for cardiac failure or hypothyroidism (*P* = 0·038 and *P* = 0·760, respectively) (Figure 1a,b). Grouping by IH number, two potentially interesting findings were: there were no cases of single IH with cardiac failure; and there was a significant difference in cardiac failure rate between those with 1–9 IH and those with 10 or more, but not between 1–4 and 5–9 (Figure 1c). However, the total number of cases with adverse clinical outcomes was very small, and these attempts to answer question (i) may still be underpowered.

**Figure 1:**
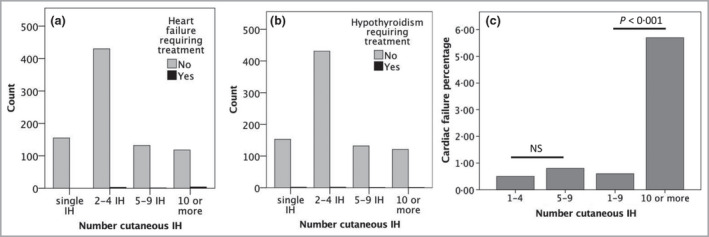
Frequency of (a) cardiac failure requiring treatment and (b) hypothyroidism requiring treatment demonstrating cases with fewer than five infantile haemangiomas (IH) in both groups who would not be detected by current recommendations for screening; (c) percentage of cases of cardiac failure requiring treatment by grouped number of cutaneous IH, demonstrating lack of significance between 1–4 and 5–9 IH, but a significant difference between 1–9 and 10 or more (*P* < 0·001, two‐way Fisher’s exact test). NS, not significant.

All eight cases treated for cardiac failure had cardiorespiratory symptoms/signs at first presentation to medical services with IH, and treatments were given on that basis rather than on USS result (one normal, one portocaval shunt without HH, two solitary HH, three multiple, one diffuse). Furthermore, in five of eight cases treatment was deemed or demonstrated to be due to a cardiovascular cause requiring surgical correction, rather than HH, potentially reflecting the confounding influence of prematurity on both structural cardiac defects and multiple IH.

Three of six cases treated for hypothyroidism were detected by neonatal screening (two with normal USS, one diffuse HH); two of six had no Guthrie test result available (one multiple HH, one multiple‐almost‐diffuse); and one had normal Guthrie (normal USS). The incidence of congenital hypothyroidism in the UK is 0·6/1000,^6^ which is not significantly different from this study or previous reports.^3^ Therefore, retesting of thyroid function in those with multiple HH would have missed the only case definitely missed by Guthrie screening.

On the basis of these data, we suggest that all infants should have a thorough clinical assessment on presentation with any number of IH, and if there are concerns about cardiac failure or hypothyroidism should be referred that day to a specialist. If there are no concerns, parents should be made aware of the early symptoms and signs, such as lethargy and poor feeding, and have rapid access to a physician if these develop. In our practice, use of USS will now be based on signs/symptoms of cardiac failure, independent of cutaneous IH number. Our data do not support routine rescreening for hypothyroidism for those with HH; however, numbers of cases are low. Going forward, a very large prospective randomized controlled trial of infants with all numbers of cutaneous IH will be required to assess the validity of USS and/or repeat thyroid testing compared with clinical assessment.

## Author Contribution

**Caroline Mahon:** Data curation (equal); Methodology (supporting); Writing‐original draft (supporting). **Kieren McHugh:** Investigation (equal). **Neda Alband:** Data curation (supporting). **Dyanne Rampling:** Investigation (equal). **Neil Sebire:** Investigation (equal). **Ella Williamson:** Data curation (supporting). **Mary Glover:** Conceptualization (lead); Methodology (equal); Writing‐review & editing (supporting). **Veronica A Kinsler:** Formal analysis (lead); Methodology (equal); Supervision (equal); Writing‐original draft (equal); Writing‐review & editing (lead).
